# Supplementary material for: The Invisible Fraction within Melanin Capable of Absorbing UV Light and with Fluorescent Properties: Is It Lacking Consideration?
Source: Int J Mol Sci. 2024 Aug 3;25(15):8490. doi: 10.3390/ijms25158490 (PMC11313076; doi:10.3390/ijms25158490)
Supplement: Supplementary file 1 [file ijms-25-08490-s001.zip › ijms-3101746-supplementary.pdf]

The Invisible Fraction within Melanin Capable of

Absorbing UV Light and with Fluorescent Properties: Is It Lacking Consideration?

Aaliyah Flake and Koen Vercruysse

Supplemental Information

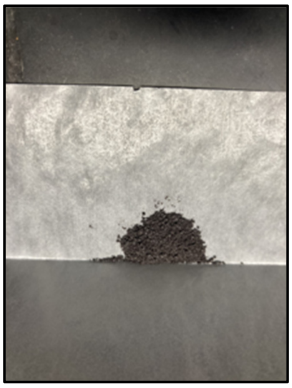

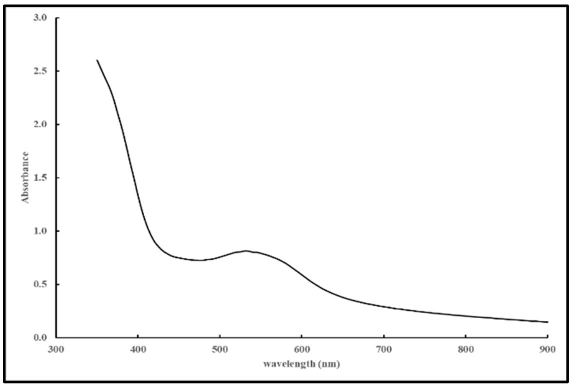

(a) **(b)**

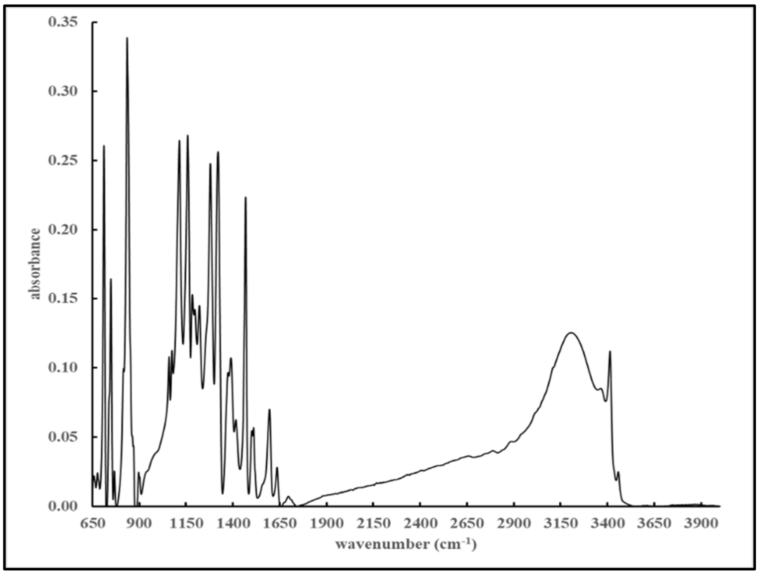

(c)

Figure S1: Evaluation of DHI precursor **(a)** Photograph of commercial DHI before reaction (photography by KV). **(b)** Absorbance spectrum of a sample containing 3mM DHI dispersed in water and centrifuged. **(c)** FT-IR spectrum of commercial DHI prior to any reaction.

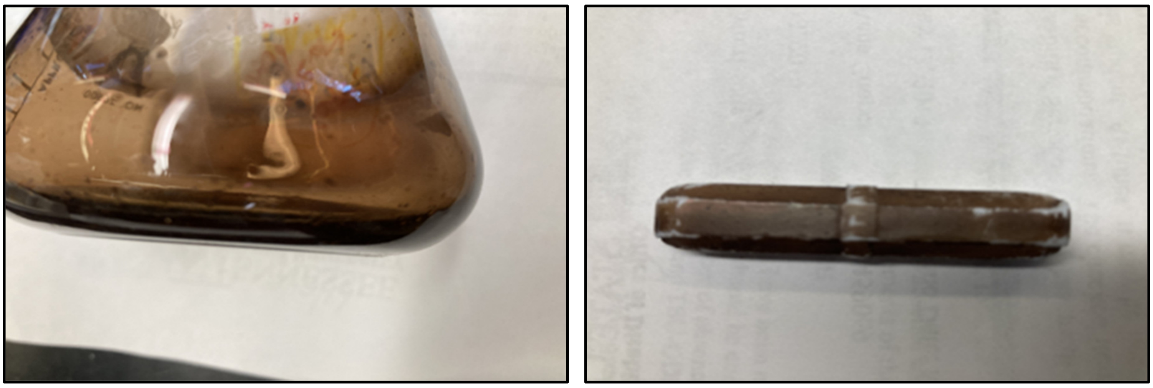

(a) **(b)**

Figure S2: Coating by serotonin-based melanin. **(a)** Glassware and **(b)** Teflon stirring bar coated with serotonin-based melanin material (Photographs by KV).

Table S1: Overview of experimental data associated with the crude reaction mixtures involving the various precursors used in this study.

Precursor	k[1]	AUC[2]	F_{disp}[3] recovered (mg)	F_{prec}[4] recovered (mg)
L-DOPA	0.0060	2,739	59	183
Dopamine	0.0059	2,199	31	196
Norepinephrine	0.0110	752	101	137
Epinephrine	0.0122	28	83	-
Catechol	0.0067	753	100	83
Pyrogallol	0.0135	1,395	75	224
3,4-dihydroxybenzoic acid	0.0141	319	133	163
Caffeic acid	0.0150	686	70	226
DHI	0.0035	5,099	-	92
serotonin	-	-	-	30

[1] Decay constant of the exponential function fitted to the absorbance spectrum of the crude mixture at the end of the reaction.
[2] Calculated (between 400 and 900nm) according to **Eq.1** following exponential regression of the absorbance spectrum of the crude mixture at the end of the reaction.
[3] See Section 2.3.
[4] See Section 2.3.

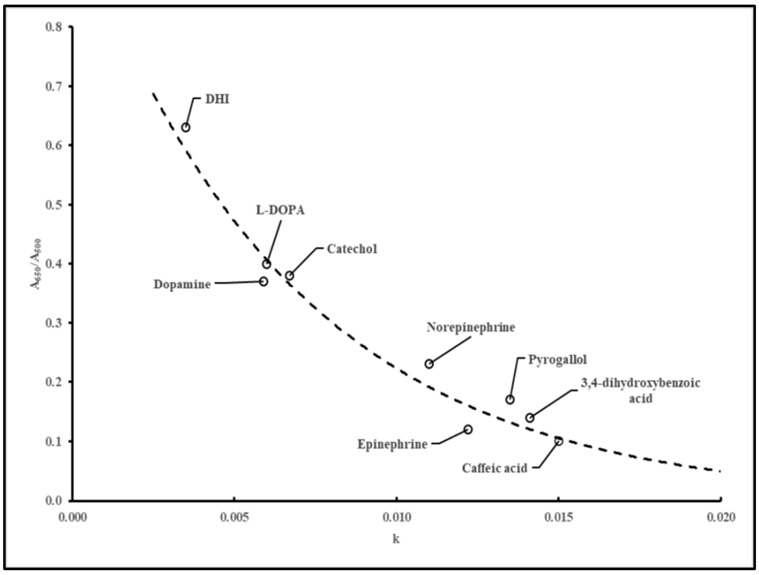

Figure S3: Relationship between the values of k and A_{650}/A_{500} (see section 4.5) obtained from the absorbance spectra of the crude reaction mixtures involving the precursors used in this study. The dotted line represents the theoretical exponential curve associated with equation 2.

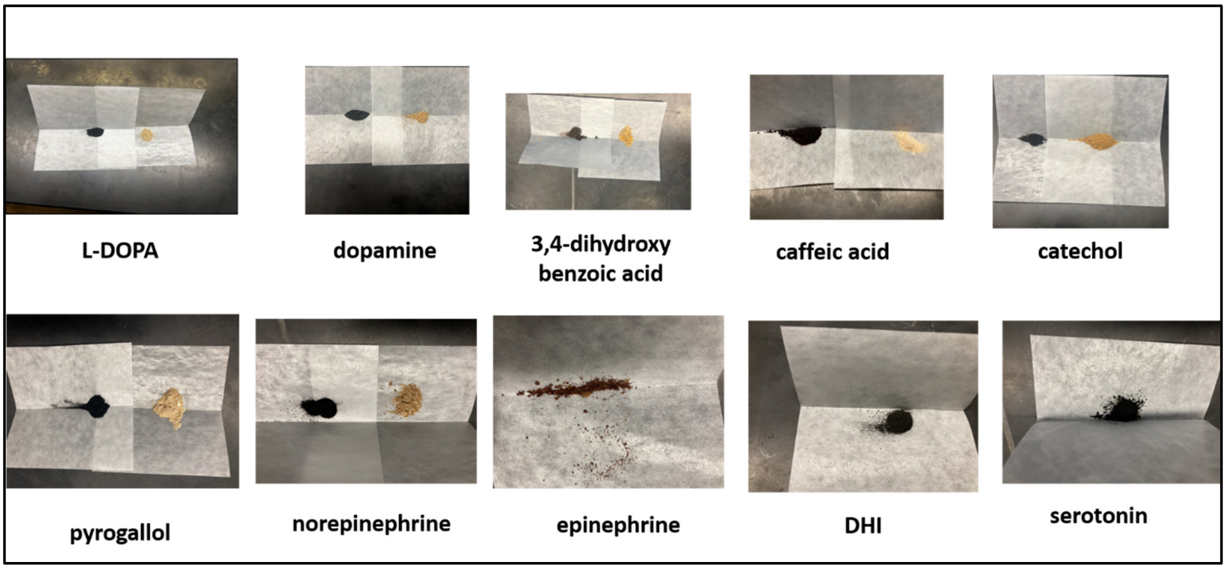

Figure S4: Photographs of the F_{prec} (dark colored) and F_{disp} (light colored) materials obtained from the various precursors used in this study. For epinephrine only a F_{disp} fraction was obtained. For DHI and serotonin only a F_{prec} fraction was obtained (Photographs by KV).

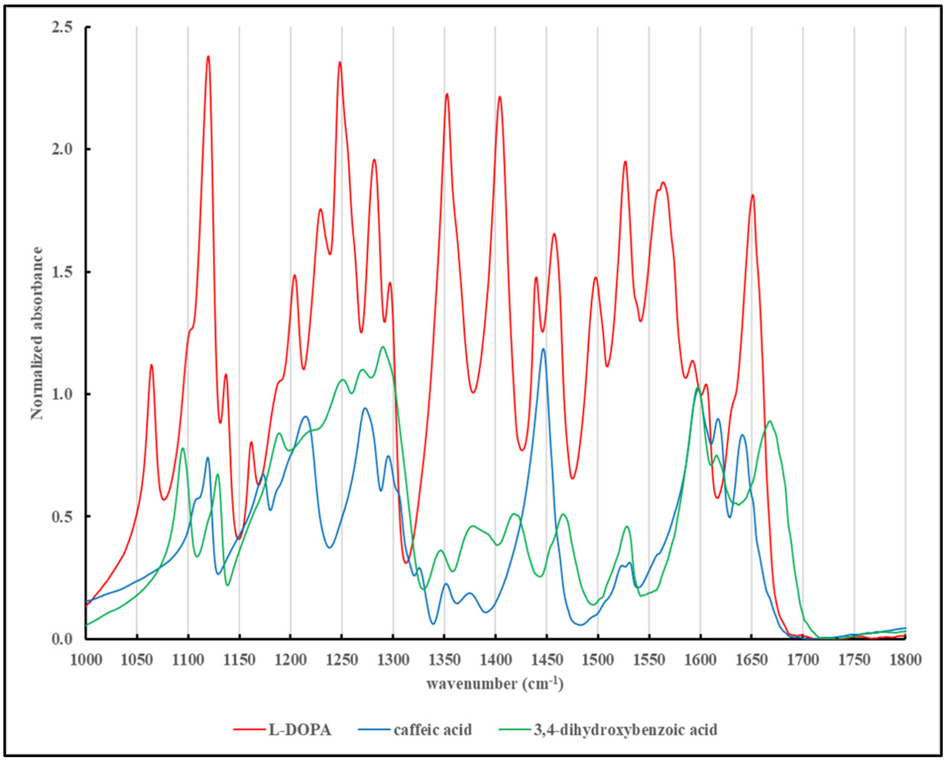

Figure S5: FT-IR spectra (portion of fingerprint region) of L-DOPA, caffeic acid and 3,4-dihydroxybenzoic acid prior to any reaction.
